# Surgical resection of a large invasive mucinous adenocarcinoma: A case report

**DOI:** 10.1002/ccr3.7707

**Published:** 2023-07-17

**Authors:** Ryusei Yoshino, Nana Yoshida, Akane Ito, Nanami Ujiie, Masaki Nakatsubo, Masahiro Kitada

**Affiliations:** ^1^ Department of Thoracic Surgery and Breast Surgery Asahikawa Medical University Hospital Asahikawa‐shi Japan

**Keywords:** IMA, invasive mucinous adenocarcinoma, lung cancer, surgery, T4

## Abstract

Invasive mucinous adenocarcinoma often presents with pneumonia‐like findings on imaging, which complicates its diagnosis. This case demonstrates that aggressive bronchoscopy is useful for examining infiltrating shadows in the lung field and large tumors occupying the entire lung lobe should be treated cautiously when lung parenchyma develops.

## 1 INTRODUCTION

Invasive mucinous adenocarcinoma (IMA) is a special subtype of adenocarcinoma according to World Health Organization classification. IMA accounts for an estimated 2–10% of all lung cancers.^1^, ^2^ Overall, 60–90% of IMA cases present with solitary nodules, mass shadows, or ground‐glass opacity lesions, but there are also cases of extensive extension with multiple nodules or diffuse alveolar shadows. Therefore, it is sometimes necessary to distinguish the lesions from benign pulmonary diseases such as pulmonary tuberculosis, sarcoidosis, lipoid pneumonia, and alveolar proteinosis.^3^, ^4^ In addition, it is often detected at an advanced stage, which makes it difficult to treat surgically; however, limited reports of such cases have been described.^2^ Here, we describe a case of IMA that was detected preoperatively as an infiltrative shadow over the entire right lower lung lobe and treated with surgical resection along with a review of the literature.

## 2 CASE REPORT

The patient was a 74‐year‐old woman whose chief complaint was a cough that had persisted for several weeks and had first appeared around 2019. Chest radiography revealed an infiltrating shadow in the right lower lung field, and a computed tomography (CT) scan of the chest revealed frosted to infiltrative changes extending into the right lower lobe. A thorough examination was performed. Imaging findings were suggestive of lung cancer or pneumonia; however, a transbronchial lung biopsy revealed a diagnosis of lung adenocarcinoma, for which the patient was referred to our department for surgical treatment.

The patient's medical history included hypertension, a uterine myoma (after total hysterectomy), dyslipidemia, and osteoporosis. The patient had no significant family history. She had smoked five cigarettes per day for 20 years and had a Brinkman Index of 100. She was 159 cm tall, weighed 48 kg, and had a body mass index of 19.0. On physical examination, no superficial lymph nodes, including cervical lymph nodes, were palpable. The lung sounds were slightly decreased in the right lower lung field. No heart murmurs were identified. Blood samples showed no abnormalities in blood count, biochemistry, or coagulation. The tumor marker carcinoembryonic antigen level (2.1 ng/mL) was within the normal range, but the cytokeratin fragment level (10.6 ng/mL) was high. Respiratory function tests showed a vital capacity (VC) of 2650 mL, %VC of 104%, forced expiratory volume in 1 s (FEV_1_) of 1830 mL, and FEV_1_% of 75%. Electrocardiography revealed no abnormalities.

**FIGURE 1:**
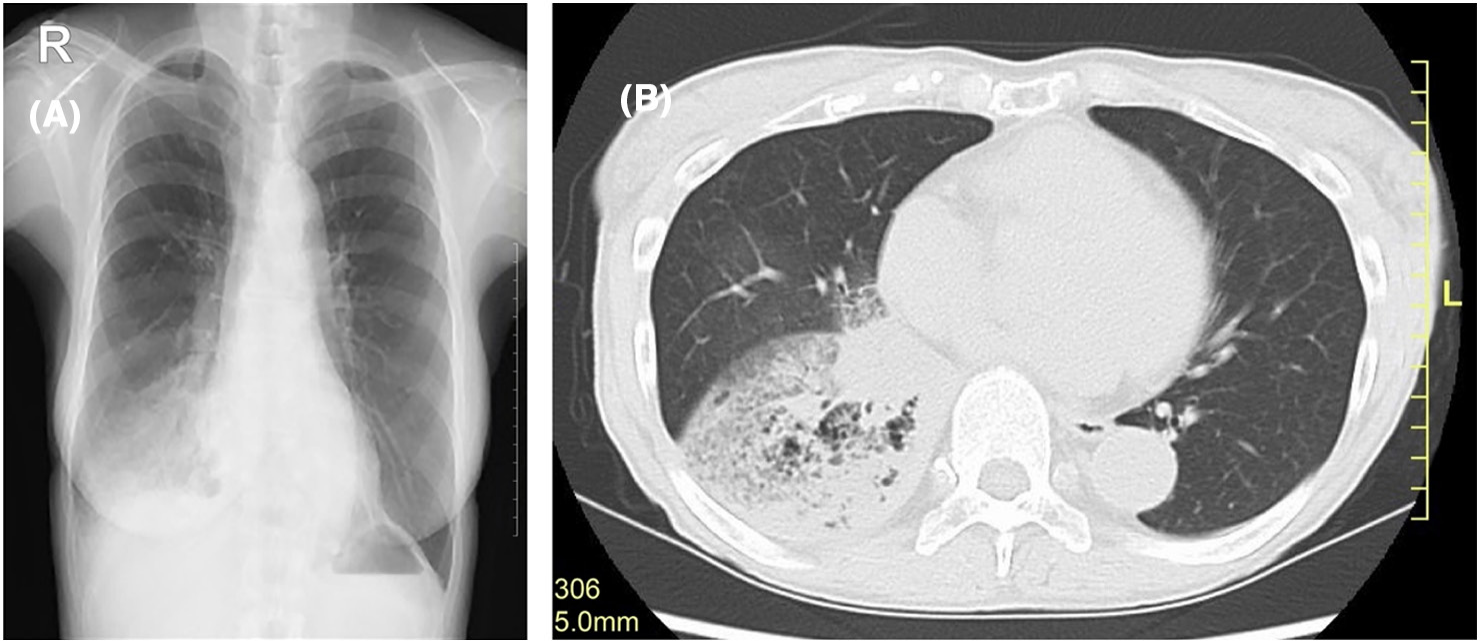
Chest radiograph (frontal view) and chest computed tomography findings. (A) An extensive infiltrative shadow is visible in the right lower lung field. (B) The entire right lower lobe had frosted to infiltrative changes extending 20 cm. Some bronchiectatic dilatations are visible in the inner part of the lobe, suggesting the possibility of middle lobe infiltration beyond the major fissure.

Chest radiography revealed an infiltrative shadow in the lower right lung field (Figure 1A). Thoracoabdominal CT showed a 20‐cm‐long frosted to infiltrative change over the entire right lower lobe with some bronchiectasis in the interior suggesting the possibility of middle lobe involvement beyond the major fissure (Figure 1B). No obvious enlargement of the peribronchial lymph nodes was observed. [^18^F]Fluorodeoxyglucose–positron emission tomography (FDG‐PET) showed a massive cavitary maximum standard uptake volume of 8.4 accumulation in the lower lobe of the right lung. The surrounding consolidation was also highly concentrated, which was consistent with malignancy. In contrast, there was no obvious accumulation in the interlobular septal wall or frosted shadows of the lower and middle lobes, nor were there metastatic findings in the other organs.

Bronchoscopy revealed no apparent abnormalities in the visible range of the bronchial lumen. A transbronchial lung biopsy and histological examination were performed on the area with a strong infiltrative shadow. Histopathological findings were adenocarcinoma, epidermal growth factor receptor (*EGFR*)–negative, anaplastic lymphoma kinase–negative, and programmed death ligand 1 Tumor Proportion Score 60%. The preoperative diagnosis was right lower lobe lung adenocarcinoma (cT3N0M0, cStage IIA). Considering the possibility of pleural dissemination and unresectability in cases of invasion into the middle lobe, thoracoscopic‐assisted right lower lobectomy was performed.

**FIGURE 2:**
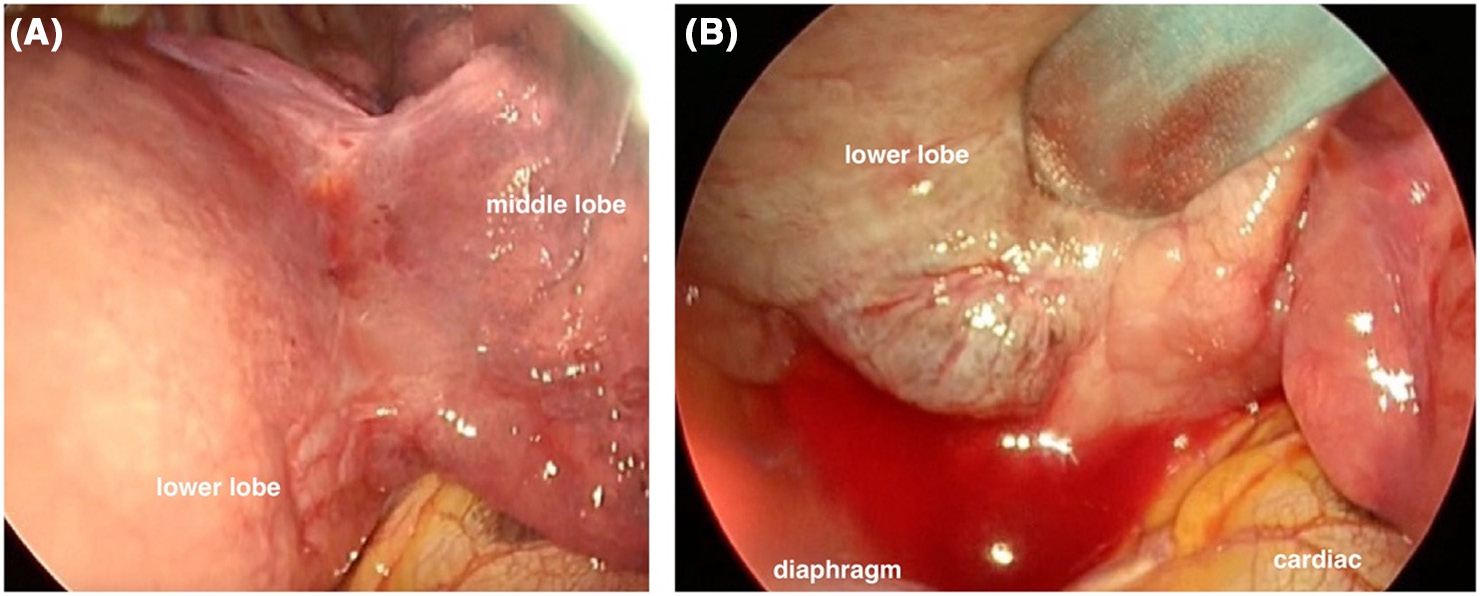
Intraoperative findings. (A) No invasion of the diaphragm, upper lobe, or middle lobe or adhesion between the pleura and the wall was noted. (B) A hematogenous pleural effusion was observed.

The patient was placed in the left lateral recumbent position under general anesthesia. Under thoracoscopic guidance, a 10‐cm skin incision was made in the fifth intercostal space and a small thoracotomy was performed. No pleural dissemination was observed. No invasion of the diaphragm, upper lobe, or middle lobe was observed. There was no adhesion between the pleura and the pleural wall. A hematogenous pleural effusion was observed (Figure 2A,B). The basilar artery, A6, and A4 + 5 were easily identified. The basilar artery and A6 as well as the pulmonary veins were separated using an automatic suturing machine. The peribronchial area was trimmed, and the bronchus was separated using the automatic suturing machine. A fat pod with a stalk of pericardial adipose tissue was placed over the bronchiectomy site. Leakage test results were uneventful. The operation time was 1 h 20 min, and the blood loss was 10 mL.

**FIGURE 3:**
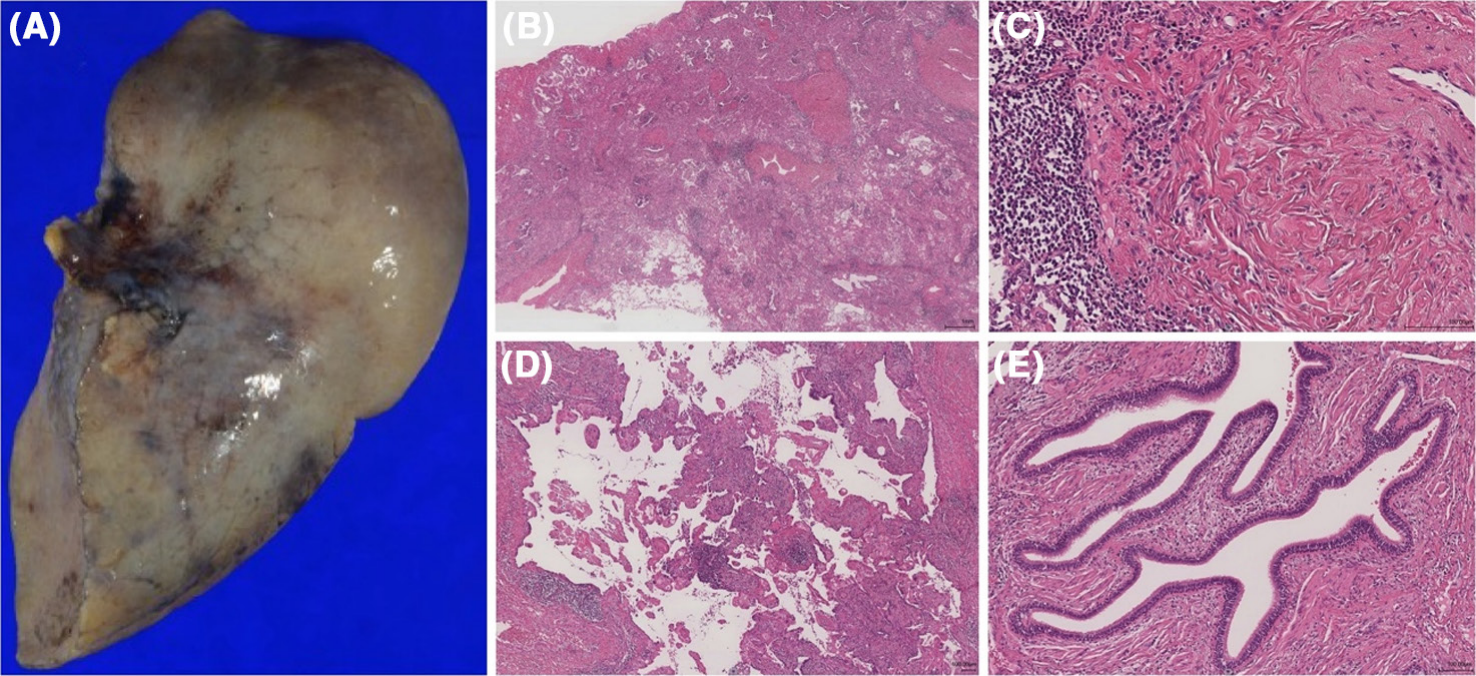
Histopathological findings. (A) The resected specimen of the lower lobe of the right lung was 19 × 9.5 × 6 cm. (B) Atypical cells proliferating in a papillary fashion were observed (hematoxylin–eosin [HE] staining, ×4). (C) Mucus accumulation in the entire lower lobe (HE staining, ×20). (D) Destruction of existing alveolar structures was noted (HE staining, ×20). (E) An irregularly fused glandular duct with lymphocytic infiltration and fibrous stromal growth were noted (HE staining, ×20).

The resected specimen of the lower lobe of the right lung measured 19 × 9.5 × 6 cm (Figure 3A). The cracked surfaces showed mucus retention. The specimen was partially dragged by the pleura, and an induration was palpable in the same area. A histopathological examination revealed mucus accumulation in the entire lower lobe centered on the papillary proliferation of atypical cells (Figure 3B,C). Destruction of existing alveolar structures was observed (Figure 3D). The patient exhibited irregularly fused glandular ducts with lymphocytic infiltration and fibrous interstitial growth (Figure 3E). A diagnosis of IMA, max 19.0 cm, pT4N0M0, pStage IIIA, was made. No venous or vascular invasion was observed. The resection margins were negative.

The patient's postoperative course was uneventful, and she was discharged from the hospital on the 8th day. After discharge, the patient received chemotherapy with carboplatin and paclitaxel as postoperative adjuvant therapy at the respiratory medicine department of another hospital. However, a right pneumothorax and bilateral lung metastases occurred during the third course of chemotherapy, and the efficacy of the treatment was evaluated as progressive disease. Therefore, chemotherapy was stopped mid‐course and the patient was started on pembrolizumab in 2019. During the first year of treatment with pembrolizumab, the patient achieved a partial response, but treatment was terminated at the 14th course due to liver damage. The patient then indicated that he wished to discontinue treatment, and it was terminated midway through the course. Thereafter, careful follow‐up was continued, and to date, the patient has remained in partial response and no new recurrence or metastasis has occurred.

Written informed consent was obtained from the patient for the publication of his case.

## 3 DISCUSSION

IMA originates from goblet cells (mucus‐producing cells) and is classified as a unique type of lung adenocarcinoma. Together with colloid adenocarcinoma, fetal adenocarcinoma, and intestinal adenocarcinoma, it is one of four subtypes of special invasive adenocarcinoma, a group conventionally called mucinous bronchioloalveolar carcinoma.^1^, ^5^, ^6^ An estimated 60–90% of cases are localized, with solitary nodular or mass shadows, while 10–40% are extensive, with multiple nodular shadows or thick consolidations. These cases must be differentiated from those of tuberculosis, alveolar proteinosis, lipoid pneumonia, sarcoidosis, and other diseases. The lower lobe is the most common site of occurrence. An important sign of differentiation is the presence of clear vascular shadows in homogeneous consolidations on contrast‐enhanced CT. FDG‐PET is reportedly useful for differentiating IMA from other diseases such as pneumonia.^3^, ^4^, ^7^ The present case is significant because there are relatively few reports of surgical resection of IMA detected as large tumors.

This case demonstrated the usefulness of aggressive bronchoscopy for infiltrating shadows in the lung field. In this case, the patient was initially suspected as having pneumonia because of the presence of an infiltrative shadow in the right lower lobe the diagnosis of a coughing patient. However, a definitive diagnosis of lung adenocarcinoma was made on transbronchial lung biopsy. Although the prognosis of IMA is generally good, cases with pneumonia‐like findings on chest CT reportedly have a poor prognosis because the histopathological stage is often high owing to the large tumor size.^8^ In contrast, IMA detected at an early stage with a tumor diameter of less than 3 cm reportedly shows a good prognosis after surgery.^9^ This case suggests that aggressive bronchoscopy is useful for patients with infiltrating shadows in the lung field without acute inflammatory symptoms with the tumor in mind.

Careful treatment of the lung parenchyma is important when a large tumor occupies the entire lung lobe, as in the present case. Large tumors that occupy the entire lung lobe are often difficult to collapse, and there is a risk of mucus leakage due to injury to the visceral pleura by forceps, which can cause the seeding of malignant cells. In addition, excessive compression of the removed lung can also cause visceral pleural damage; therefore, an open chest wound is necessary depending on the tumor diameter and volume. Thoracoscopic surgery is becoming increasingly common. Therefore, some reports concluded that video‐assisted thoracoscopic surgery for primary lung cancer with a tumor diameter of >5 cm should be performed with caution to prevent cancer cell loss. However, the authors were of the opinion that care should be taken to avoid tumor damage in such cases.^10^ In this case, considering the tumor's size and the fact that the entire lung lobe was stretched by mucus, surgical safety using a 10‐cm skin incision was considered best.

Recent studies identified mutations in the *KRAS*, v‐raf murine sarcoma viral oncogene homolog B1, v‐erb‐b2 erythroblastic leukemia viral oncogene homolog 2, and phosphatidylinositol‐3 kinase catalytic alpha genes as well as other genetic abnormalities. Genes harboring rare mutations were also identified. Among them, *KRAS* mutations are frequent, whereas *EGFR* mutations are rare.^11^ This finding supported the hypothesis that many patients respond poorly to cytotoxic drugs. Other studies demonstrated that oncogenic fusions act as driver mutations for IMA in *KRAS*‐negative cases and, thus, may be promising therapeutic targets for IMA.^12^ These studies broadened the scope of gene therapy for IMA, and there is a need for the further development of therapeutic agents.

## 4 CONCLUSION

Here, we reported a case of large IMA that required preoperative differentiation from pneumonia. This case illustrates the following two points. First, aggressive bronchoscopy is useful for detecting infiltrating shadows in the lung fields. Second, a large tumor occupying the entire lung lobe requires careful handling due to the development of the lung parenchyma. It is currently important to aim for complete surgical resection to ensure safety, even if the tumor is larger than T3 preoperatively.

## INSTITUTIONAL REVIEW BOARD STATEMENT

The study was conducted in accordance with the guide‐lines of the Declaration of Helsinki and was approved by the Institutional Review Board of Asahikawa Medical University Hospitalcv (No. 22150). Approval date: March 10, 2023.

## AUTHOR CONTRIBUTIONS

**Ryusei Yoshino:** Conceptualization; writing – original draft. **Nana Yoshida:** Writing – review and editing. **Akane Ito:** Writing – review and editing. **Nanami Ujiie:** Writing – review and editing. **Masaki Nakatsubo:** Writing – review and editing. **Masahiro Kitada:** Supervision; validation; visualization.

## FUNDING INFORMATION

This research received no external funding.

## CONFLICT OF INTEREST STATEMENT

The authors declare no conflict of interest.

## CONSENT

Written informed consent was obtained from the patient to pub‐lish this paper.

## Data Availability

Not applicable.
